# Current good manufacturing practice considerations for mesenchymal stromal cells as therapeutic agents

**DOI:** 10.1016/j.bbiosy.2021.100018

**Published:** 2021-05-05

**Authors:** Clara Sanz-Nogués, Timothy O'Brien

**Affiliations:** Regenerative Medicine Institute (REMEDI), CÚRAM, Biomedical Science Building, National University of Ireland, Galway, Ireland

**Keywords:** Mesenchymal stromal cells, Cell therapy, Good manufacturing practice, Clinical grade

## Abstract

Producing human mesenchymal stromal cells (MSCs) for clinical use requires adherence to current good manufacturing practice (cGMP) standards. This is necessary for ensuring standardization and reproducibility through the manufacturing process, but also, for product quality and safety. However, the large-scale production of clinical-grade MSCs possesses unique regulatory challenges and hurdles related to the heterogeneous nature of MSC cultures as well as the complex manufacturing process. Following is a compilation of the major issues encountered in the manufacturing of MSCs for clinical use, and our views on the optimal characteristics of the final MSC product.

## Highlights

• MSCs are of interest to the medical and scientific community due to their immunosuppressive and regenerative properties.
• The large-scale production of MSCs possess unique regulatory and manufacturing hurdles.
• Here, there is a list of the major cGMP considerations and challenges that need to be addressed when designing MSC therapies.

## Introduction

Advanced therapy medicinal products (ATMPs), including cell, gene and tissue-engineered therapies, offer groundbreaking new opportunities for the treatment of conditions of unmet medical need. Mesenchymal stromal cells (MSCs) have gained enormous attention across the medical and scientific community due to their potent immunosuppressive and regenerative properties [1]. During the past years, the safety and efficacy of this cell therapy product has been investigated across the world for a wide variety of clinical indications [1]. However, the MSC pathway to the clinic has not been straightforward and has challenges not encountered in the traditional pharmaceutical industry.

In Europe, MSCs are considered an ATMP, in particular, a somatic-cell medicinal product, and therefore it is governed by a specific ATMP regulatory framework, the Regulation 1394/2007/EC and Directive 2009/120/EC. This regulation provides specific principles for the evaluation and authorization of ATMPs in the EU. MSCs will also be regulated by the guidelines of medical devices, Regulation (EU) 2017/745 and Regulation (EU) 2017/746, when used in a combinatorial fashion. In addition, the production of human MSC doses for clinical use should be performed under strict adherence to European current good manufacturing practice (cGMP) guidelines (EudraLex Volume 4, Part IV). The ultimate goal of these complex regulations is to ensure patient safety and well-being.

**Fig. 1:**
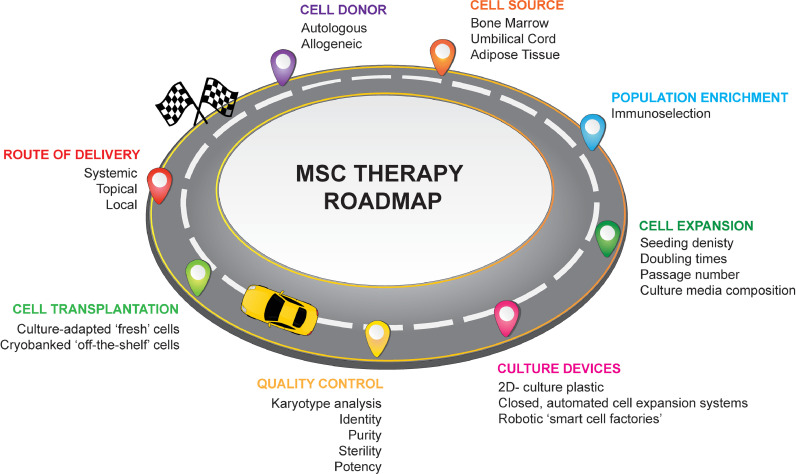
Mesenchymal stromal cell roadmap showing key steps in the manufacturing process.

However, complying with cGMP standards requires a precise and well-defined product with a cell manufacturing roadmap, from the moment of cell acquisition and isolation to culture expansion and transplantation at the bedside (Fig. 1). While there has been considerable success in manufacturing MSCs at laboratory scale, less consideration has been afforded to how these technologies can be translated on a global scale.

EU regulation of ATMPs is onerous and constantly evolving, resulting in a complex regulatory environment across the 27 EU member states, and it represents a major bottleneck for progress in the field. Nevertheless, regulatory centralization has been introduced for ATMP marketing in the EU with recent updated guidelines for a Voluntary Harmonization Procedure (VHP) for the assessment of multinational clinical trial applications (CTFG/VHP/Rev 7, October 2020). This initiative was created to harmonize the design, development, manufacture and authorization of ATMPs in the EU and speed up the process of multinational clinical trial applications across the EU member states.

But apart from the bureaucratic difficulties, MSC-specific manufacturing hurdles also exist. These result from the heterogeneity of MSC cultures, often manifest at the level of MSC donors and tissue sources [2] but also, due to the current lack of standardization and harmonization of MSC manufacturing protocols. Single alterations in the bioprocess have the potential to change the final product, therefore, having consistent and reliable manufacturing procedures, with full control of all the process variables, is essential for ensuring quality and safety. In addition, reproducibility across cell manufacturing processes would allow for comparability of MSC safety and efficacy profiles across different clinical studies, something which is currently quite difficult to achieve.

To date, both, public institutions (academic, hospitals) and the private sector (small medium enterprise (SEM) or big companies) are involved in the development of novel ATMPs. Nevertheless, the challenges associated with the therapeutic agent's GMP scalability, as well as the risk-benefit assessment may differ greatly between the two sectors. Despite all the scientific and regulatory challenges encountered by academic institutions, they have managed to bring novel therapeutics into early-phase clinical trials using public economic resources, often to test treatment of no-option or low-incidence diseases. However, academic institutions, and also SMEs, encounter great difficulty in moving to late-phase studies due to a lack of personnel, infrastructure and capital. On the other hand, large private companies may have resources for scale-up strategies and long-term economic sustainability, but they may find a high-risk approach to invest in novel cell-based therapeutics for diseases with low incidence, of autologous nature, or when there is still a lack of complete understanding of the product's mechanism of action (MoA). However, instead of seeing academia and industry as separate sectors, partnership models, by means of specific agreements, will more likely be the most effective and safer path towards ATMP marketing. This partnership model can share the knowledge, skills, and resources but also, the risks involved especially during the early and most uncertain phases of ATMP development.

Overall, the production of clinical-grade MSCs requires a critical review of the entire manufacturing process (Fig. 1). Efforts at harmonizing these processes will result in an optimized MSC therapeutic product(s) and culture conditions that can be widely used for patient benefit. In this article, the authors have compiled a list of the major cGMP considerations and current challenges to be addressed in order to achieve safe, consistent and affordable MSC therapies applicable worldwide.

**Top 10 cGMP considerations when developing MSC therapeutics.**

(1) *MSC donor.* MSCs can be obtained from patient's own cells (autologous) or from other donors (allogeneic). Autologous therapies possess important logistic hurdles, despite the advantage of removing concerns with potential donor-specific immune reactions. This is especially important for acute or rapidly progressive diseases such as sepsis, stroke, myocardial infarction, or critical limb ischemia (CLI), where delays during the cell manufacture and quality control testing would render the clinical applicability of these autologous cells unsuitable for these patients [3]. In such cases, an allogeneic ‘of-the-shelf’ MSC product may be seen as a more rational model. The rationale behind this approach is that MSCs have been shown to be hypo-immunogenic, and certainly have not been seen to mount a strong immune response when delivered in allogeneic settings. However, it is now reasonable to acknowledge that allogeneic MSCs do indeed trigger a donor-specific immune response *in vivo*, an observation which should be considered when using allogeneic MSC therapies [4]. Other factors such as gender, age, disease severity, co-morbidities and/or clinical history of donors should also be taken into consideration. For instance, there is increasing evidence of MSC gender-effects on differentiation potential, proliferation, secretome and therapeutic efficacy [5]. On the other hand, age and/or health status of the donor have been shown to impact MSCs properties [6] and may also be related to the appearance of karyotypic abnormalities [3]. However, whether the appearance of these karyotypic abnormalities is intrinsic to these older/diseased cells or if this occurs during *ex vivo* expansion of cells, is not yet well understood.
(2) *Cell source*. MSCs can be obtained from multiple different adult tissue sources but, most commonly, they have been isolated from bone marrow (BM), umbilical cord and adipose tissue. It is now increasingly recognized that the regenerative potential of these MSC-like cells may be contingent upon the tissue source, and therefore, a prominent question remains, whether individual medical conditions would benefit from a specific cell source. Nevertheless, the choice of tissue MSC sourcing, as well as the methods for cell isolation may often be driven by intellectual and/or industrial property reasons in addition to issues of biological superiority or other scientific reasons.
(3) *MSC expansion characteristics*. Upon isolation, MSCs have to undergo extensive *in vitro* expansion in order to achieve clinical doses. Factors such as isolation procedure, plating cell density, doubling times, number of passages and confluency have important effects on MSC growth kinetics and performance. Paradoxically, these aspects have not yet been standardized across laboratories. Currently, the effect of the *in vitro* expansion on the characteristics of these cells is not well understood, and therefore, regulators demand cell karyotypic analysis for batch release. However, there is still no consensus as to the minimum standards for quality control that are required for the GMP production of MSC therapeutic agents [7].
(4) *Culture media*. To date, the majority of laboratories have used fetal bovine serum (FBS) as a media supplement to expand MSCs, but this is not a future viable option. FBS content is not well-defined, and it presents a significant risk of inter-species cross-contamination. Alternatives include human platelet lysate (hPL), but the potential risk of disease transmission and its limited availability represent bottlenecks for large-scale production. Alternatively, new GMP-compliant, commercially available, chemically well-defined xenogeneic-free media that support MSC growth would constitute a more cost-effective and risk-reduced approach. Although these new formulations may influence MSC phenotype and performance, when successful, they will have the potential to enhance batch-to-batch consistency in the cell manufacturing process.
(5) *MSC fitness*. MSC therapeutics have been delivered at the bedside as culture-adapted or ‘fresh’ cells, with optimal metabolic fitness and high replication capacity, or cryobanked ‘off-the-shelf’ cells that are thawed immediately prior to transplantation. While the first approach has important logistic problems, thawing after cryopreservation has been shown to have significant short-term effects on MSC viability, functionality and *in vivo* persistence [8]. Although these effects can be reverted within 24 h following reestablishment of cell culture, the vast majority of human clinical trials administer MSCs that are thawed immediately prior to transplantation, where MSCs are unlikely to have reverted the effects of cryopreservation. Thus, cryobanked cells could be considered to be less optimal than metabolically fit culture-adapted cells. Nevertheless, efferocytosis, or engulfment of apoptotic MSCs by phagocytic macrophages, is an alternative theory that may explain MSC-mediated immune suppression [9], in which case the concept of MSC fitness may become less relevant. In any case, when an ‘off-the-shelf’ approach is preferred, the use of cryoprotectant formulations which are xenogeneic-free, chemically defined, dimethyl sulfoxide (DMSO)-free, and that can be delivered without further manipulation at the bedside, are highly desirable.
(6) *MSC population enrichment*. MSC cultures are heterogeneous in nature [2]. An individual surface marker that is truly MSC-specific does not yet exist, and in most cases, isolation of these cells relies on plastic adherence. Cell enrichment by prospective immunoselection has been proposed as an alternative technology to obtain more homogeneous, well-defined and pure MSC products. Selection of cells is based on the use of specific antibodies that are directed against specific cell surface markers. These cells can then be purified using cell sorting technologies. In this context, a sub-population of stromal cells and mesenchymal progenitor cells (MPCs) have been isolated using antibodies against Syndecan-2 (CD362) [10] and stromal precursor antigen-1 (STRO-1) [11], respectively. While this technology offers advantages such as enhancing product purity and consistency, it introduces additional steps in the manufacturing process, and thus it requires additional cGMP grade-compatible reagents and technologies, as well as additional safety and quality control mechanisms to be in place.
(7) *Large-scale culture devices*. MSCs have been traditionally expanded using 2D-culture plastics, but this is extremely time-consuming and labor-intensive. Alternatively, a wide range of closed, automated, high-volume cell expansion system are currently available in the market for cell therapy product manufacturing, which offer great advantages [12]. While initial studies must be performed to ensure that MSC phenotype and performance is not affected, it offers important benefits such as the possibility of accurate and real-time measurement of processing variables such as pH, dissolved oxygen_,_ metabolite accumulation or contaminants, which will ultimately enhance product consistency while meeting safety and quality standards.
(8) *Global-scale MSC production*. Ultimately, the use of cutting-edge, automated, robotic ‘smart cell factories’ for industrial-scale and global production of MSC therapeutic doses will be necessary [13]. This will increase safety and reproducibility during the manufacturing process but ultimately will reduce the production times and costs and will generate more affordable therapies. Nevertheless, important challenges are anticipated such as significant capital investment for building state-of-the-art infrastructure, new quality and safety standards, regulatory harmonization across countries, and successful inter-sectoral and academic-industry partnership.
(9) *Quantifiable metrics for predicting MSC therapeutic efficacy*. Potency of a product can be defined as a ‘quantitative measure of relevant biological function based on the attributes that are linked to relevant biologic properties’ [14]. Potency assay(s) may consist of one or more bioassays (*in vitro* or *in vivo*), and/or non-biological analytical assay(s) that use surrogate measurement(s) that have been correlated to a product-specific biological activity [15]. They are key for ensuring quality, consistency and stability of the manufactured product, and as such are used for batch release, which must be fully validated in phase III clinical studies. They are also used as functional predictors of product effectiveness in a given clinical indication, and thus, their design must be informed by the product's MoA. This is particularly challenging for MSC therapeutics, as the MoA underlying MSC clinical efficacy is not yet fully understood, although recent reports have shed some light on this topic [16]. In addition, while the public disclosure of functional markers of MSC potency for specific clinical applications would help to advance the field, this may be restricted due to intellectual property.
(10) *Combined ATMP approaches*. The next generation of MSC therapeutics will consist of complex ATMPs that may include a combination of cell therapy products, genetic engineering products (viral and/or non-viral vectors), tissue engineering products (biomaterials/scaffolds), tissue architecture techniques (3D bioprinting and decellularized organs) and/or medical devices. While it is definitely an exciting future, these approaches will be complex, both in terms of GMP scalability and regulatory aspects. In addition, quality assurance in the bioprocess is a primordial primary consideration for ensuring the safety of these complex medicines, which by nature may be personalized to each patient.

### Expert opinion

In the past 15 years, the Regenerative Medicine Institute (REMEDI) at National University of Ireland, Galway, has moved research performed at laboratory scale to clinical trials. A crucial enabler of this translation was the construction of a GMP facility by the University and successful licensing of the Centre for Cell Manufacturing Ireland, as well as funding provided by the EU Commission for the clinical trials. Principal Investigators at REMEDI are now conducting early-phase clinical trials using MSCs for treating different clinical conditions including osteoarthritis, CLI, diabetic nephropathy and corneal transplantation (Table 1). The path to the clinic has been challenging and we have encountered regulatory and GMP-related hurdles associated with the issues outlined above. Our strategy for cell manufacturing and choice of therapeutic product continues to evolve conscious of these issues. Our first clinical trial utilized autologous BM-MSCs, culture-expanded in 2D-culture flasks using FBS and delivered intramuscularly as a cryobanked product, with a wash of the cryopreservative prior to delivery to patients at an approved cell manufacturing site in the hospital. We have reported on the challenges of this autologous approach and suggested allogeneic approaches may be preferred for CLI [3]. Since then, other trials at REMEDI have used cells from different tissue sources, such as adipose tissue, and has utilized allogeneic ‘off-the-shelf’ BM-MSC sources for other clinical indications. REMEDI has partnered with Orbsen Therapeutics Ltd. to undertake a clinical trial with their CD362 enriched BM-stromal cell product (Orbcel-M^TM^) in patients with diabetic nephropathy. We have moved from using FBS supplemented media to using xenogeneic free alternatives such as hPL. We also have expanded cells using a large-scale, closed, automated culture expansion system such as the Quantum® Cell Expansion System [17]. Our plan for the future is to harmonize the whole MSC manufacturing process for different clinical conditions to the extent possible, and to work towards a unique allogeneic ‘off-the-shelf’ MSC product, ideally obtained from freely available tissue sources such as umbilical cord tissue, which require non-invasive procedures for cell isolation; cultured in GMP- and regulatory-compliant xenogeneic-free media, expanded in a closed automated bioreactor system and delivered ‘off-the-self’ as a cryobanked product suspended in a chemically-defined, DMSO-free media, which do not require further manipulation at the bedside. Finally, in the future, we would aim to use automated robotic factories such as those being developed by colleagues in the AUTOSTEM EU consortium [18]. Nevertheless, a current challenge is the need to repeat costly pre-clinical safety and efficacy studies when changes are introduced in the bioprocess.

**Table 1:** Early-phase clinical trials undertaken by Principal Investigators at REMEDI.

Disease condition	Phase	MSC source	MSC Donor	Culture device	Media supplements	MSC delivery method	Clinicaltrial.gov ID
Critical limb ischemia	1b	BM-MSCs	Autologous	2D-culture flasks	FBS	Cryobanked cells with a wash step	NCT03455335
Osteoarthritis	2	ASCs	Autologous	2D-culture flasks	hPL	Culture-adapted cells	ADIPOA-2 NCT02838069
Diabetic nephropathy	1/2	CD362+ BM-MSCs	Allogeneic	Quantum cell expansion system	hPL	Cryobanked cells	NEPHSTROM NCT02585622
Cornea transplant	1b	BM-MSCs	Allogeneic	2D-culture flasks	hPL	Cryobanked cells	VISICORT n/a*

ASC=adipose derived stem cells.; BM=bone marrow, MSC=mesenchymal stromal cells; FBS=fetal bovine serum; hPL=human platelet lysate. n/a* VISICORT Trial does not have a clinicaltrial.gov identification number yet but the EudraCT ID is 2018-000890-60.

## Declaration of Competing Interest

TOB is a founder, director and equity holder in Orbsen Therapeutics Ltd. CSN declares no conflict of interest.
